# Root coverage in Miller’s Class I and II gingival recession using acellular dermal matrix and subepithelial connective tissue graft: A systematic review

**DOI:** 10.34172/japid.025.3398

**Published:** 2025-01-20

**Authors:** Aaesha I.A Khan, Sujeet V. Khiste, Vineet Kini

**Affiliations:** Department of Periodontics, Mahatma Gandhi Mission’s Dental College and Hospital, Navi Mumbai, Maharashtra University of Health and Science, Nashik, India

**Keywords:** Acellular dermal graft tissue, Allografts, Connective tissue, Gingival recession

## Abstract

**Background.:**

Subepithelial connective tissue graft (SCTG) is considered the gold standard in the treatment of gingival recession. Donor site morbidity has led to the introduction of non-autogenous grafts such as acellular dermal matrix (ADM). This systematic review compared SCTG with ADM to treat root coverage in Miller’s Class I and Class II gingival recession.

**Methods.:**

Articles in PubMed (11), Scopus (1), EBSCO (2), and Google Scholar (1) were incorporated in this study. The studies included randomized controlled trials from 1st January 2011 to 31st December 2022. Studies that compared root coverage and reduction in recession depth using SCTG and ADM grafts were included in the review. Fourteen randomized control trials (RCTs) were included in this systematic review following the PRISMA guidelines. The risk bias assessment was assessed using the ROBINS-2 tool.

**Results.:**

Of the 14 included studies, 11 articles concluded that ADM is as effective as SCTG for treating root coverage in Class I and Class II gingival recession. However, 3 studies showed a statistically significant difference between SCTG and ADM results, indicating better root coverage achieved with SCTG than with ADM.

**Conclusion.:**

ADMs may be an alternative treatment in cases where multiple areas of recession where SCTGs harvested from the palate area would be insufficient.

## Introduction

 Displacement of the gingival margin apical to the cementoenamel junction (CEJ), leading to root surface exposure, is known as gingival recession.^1^ Various etiologic factors can result in gingival recession, such as tooth brushing trauma, restorations invading the biological width, improper brushing technique, plaque-induced inflammatory lesions, and surgical periodontal interventions. Gingival recession can lead to root hypersensitivity, root caries, and unpleasant esthetics, particularly in the anterior regions of the jaws.^2^ Subepithelial connective tissue graft (SCTG) is considered the gold standard in the treatment of gingival recessions for root coverage. It provides long-term stability of complete root coverage (CRC) in Miller’s Class I and Class II gingival recession. However, the main disadvantage of this technique is the need for a second surgical area, limited amount of graft, low patient tolerance, and high complication rates at the donor site.^3^

 To overcome the limitations, new biomaterials, such as ADM, have been introduced as an alternative to SCTGs.^4^ ADM is an allograft tissue devoid of epidermal and dermal cells. It is chemically processed to remove the cellular components while preserving the remaining bioactive dermal matrix. This matrix consists of collagens, elastin, blood vessel channels, and bioactive proteins that induce revascularization, cell repopulation, and tissue remodeling. The collagen fibrillar network allows ADM to maintain its structural integrity, thereby giving it characteristics of a feasible soft tissue graft material.^5^

 Various ADM materials are available on the market, such as Alloderm (LifeCell.Biohorizon INC), Puros dermis allograft, Perioderm, Oracell, Surederm, and Mucoderm, which can be effectively used to treat gingival recession and achieve root coverage.^5^

 Al Hamdam^6^ assessed the long-term predictability of ADM in root coverage procedure with a follow-up of three years. The study showed that ADM combined with a coronally advanced flap could improve the width of keratinized tissue and provide root coverage in teeth with gingival recession.

 Considering the various advantages of ADM, this systematic review compared root coverage in Miller’s Class I and II gingival recession using ADM and SCTG.

## Methods

 This systematic review was registered under PROSPERO (CRD42022362523) and conformed to PRISMA guidelines.^7^ The research question was based on patient/population, intervention, comparison, and outcomes (PICO). The research question of this systematic review was whether ADM (I) is as effective as connective tissue graft (C) in root coverage (O) of Miller’s Class I and Class II gingival recession (P).

 The systematic review was performed for articles published in English in Scopus, PubMed, EBSCO, and Cochrane from 1st January 2011 to 31st December 2022. The terms were combined using suitable Boolean operators (AND, OR, NOT). The keywords used were acellular dermal graft tissue, allografts, autografts, connective tissue, gingival recession, and systematic review. The search strategy used was: “gingival recession” [MeSH terms] OR (“gingival” AND “recession” OR “gingival recession” AND (“acellular dermis” [MeSH Terms] OR (“acellular” AND “dermis” OR “acellular dermis” OR (“acellular” AND “dermal” AND “matrix” [all fields]) OR “acellular dermal matrix” AND (clinical trial) OR randomized controlled trial OR systematic review.

 The inclusion criteria were randomized controlled trials comparing root coverage using ADM and subepithelial connective grafts to treat Miller’s Class I and Class II gingival recession. In vitro studies, animal models, case reports, reviews, abstracts, and unpublished articles were excluded.

 According to the inclusion and exclusion criteria, one reviewer investigated the titles and abstracts of the studies, based on which two experts screened extracted articles from the database. Disagreements were resolved by discussion. The final step included all full-text articles for the study. The main outcome was to assess whether the ADM was better in improving root coverage in Class I and Class II gingival recession compared to connective tissue grafts.

 Microsoft Word was used for organizing data extraction, which included the name of the author, year, study design, study duration, participants, parameters, surgical method, postoperative instructions, results, and conclusion. Two entries were used for blinding: (1) operators and (2) outcome assessors. Assessment risk of bias using ROBINS-2 for randomized studies and used as per Revised Cochrane risk-of-bias tool RevMan 5.4.1 for systematic review.

 Two review authors independently undertook the risk of bias assessment. Any disagreements were resolved by discussion. The outcome of the trial was noted when the operator assessed it. The response options were Yes/Probably yes/Probably no/No/No information. There was good reliability between the two reviewers with a high kappa coefficient (k > 0.89).

 The two-part tool addressed five specific domains (namely, randomization process, deviations from intended interventions, missing outcome data, measurement of the outcome, and selection of the reported result). The possible risk-of-bias judgments were assessed as low risk of bias, some concerns, and high risk of bias. Risk of bias were presented as summary review of authors’ judgments about risk of bias item for each study and overall percentages across all included studies.

## Results

**Figure 1:**
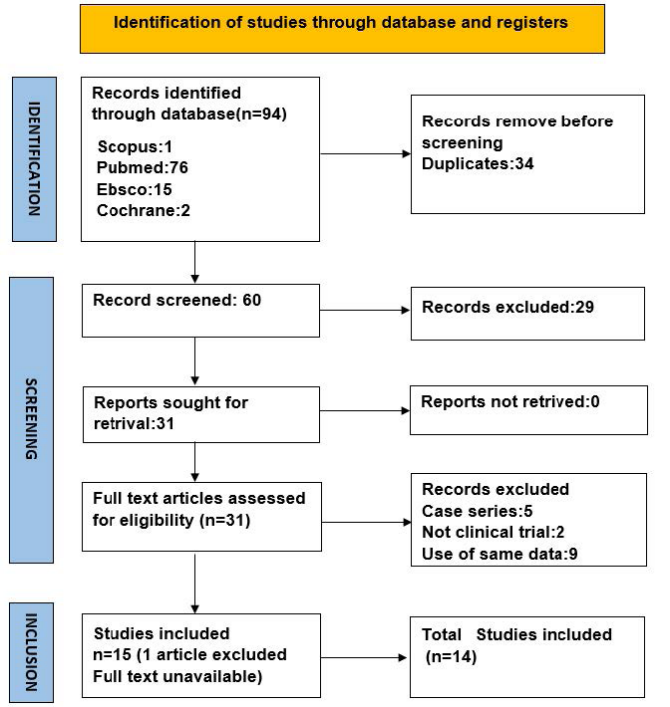
PRISMA flowchart

**Figure 2:**
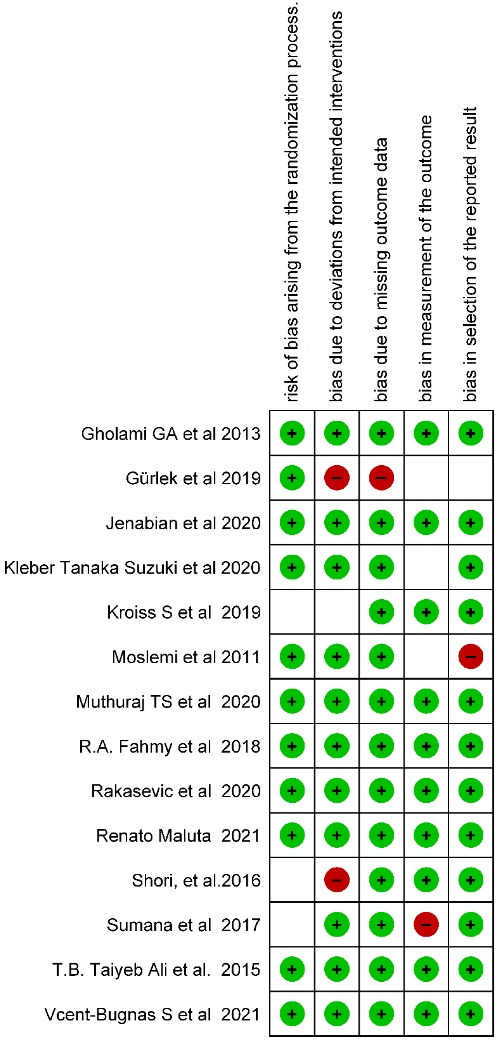
Risk of bias summary: Review authors’ judgments about each risk of /bias item for each included study

**Figure 3:**
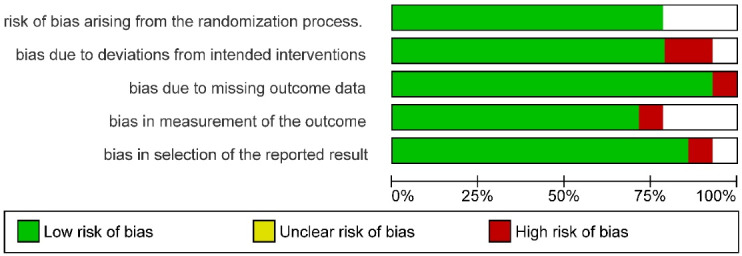
Review authors’ judgments about each risk of bias item presented as percentage across all included studies

 The systematic search resulted in 94 articles. After screening and removing the duplicates, the remaining 31 articles were included. A comprehensive assessment was conducted by the review team for the title of the articles and abstracts and after excluding the unrelated articles from the research objectives. Eventually, 15 articles were obtained after screening, out of which one was excluded due to the unavailability of the full text. Therefore, 14 articles were included, which is depicted in the form of a PRISMA screening flowchart (Figure 1). Based on the revised Cochrane risk-of-bias tool for randomized trials (RoB2) scoring system. Each included study demonstrated a low risk of bias (Figure 2). Overall the articles showed low risks of bias (Figure 3). Data obtained from the articles have been included in Table S1 (see Supplementary file 1).^8-21^ The study included a population with more than one gingival recession site. Included articles were randomized clinical trials.

 Based on the data extracted from 14 studies, 11 articles concluded that ADM is as effective as SCTG for treating root coverage in Class I and Class II gingival recession. Statistically significant results were obtained in three studies, which favored SCTG over ADM for root coverage in Miller’s Class I and Class II gingival recession.^10,17,21^

## Discussion

 The purpose of the present systematic review was to compare root coverage in SCTG versus ADM in treating Miller’s Class I and Class II gingival recession. According to Chambrone et al,^22^ the root coverage produced by ADM was similar to those achieved by SCTG. Maluta et al^17^ conducted a comparative assessment between SCTG and ADM for root coverage. A statistically significant difference (*P* = 0.045) was seen at 6 months between SCTG and ADM, with better root coverage results observed in the SCTG group.^17^ In SCTG, preexisting blood vessels anastomose with those in the gingival connective tissue. ADM, being avascular, acts as a scaffold for cells from the surrounding tissues. The collagen fibers in the ADM membrane facilitate root coverage by stimulating platelet attachment, enhancing fibrin linkage, and having a chemotactic effect on nearby fibroblasts.^23^ The crosslinking of the ADM helps increase the tensile strength, stiffens the matrix and bulk of the graft, and reduces the porosity of the matrix. As healing proceeds, ADM is fully integrated into the host tissues. It completely depends on the recipient bed, which is eventually degraded and replaced by the host cell.^24^ Because of its elastic matrices and undamaged collagen fibrils, ADM can be used as a substitute for CTG in root coverage procedures. However, the disadvantages of using ADM include lack of vascularity, longer healing time, cost of the material, and postoperative management.^24,25^ Therefore, better root coverage results were in favor of SCTG than in the test group. Inductive properties of ADM graft are determined by the percentage of colonization of non-vital graft and host cells inducing keratinization. Connective tissue grafts are entirely made up of host cells and, hence, can induce epithelium keratinization.^25^

 Owens et al^26^ reported increased resident fibroblasts and blood vessels gradually within the first few weeks and reduced inflammatory infiltrate. The graft material showed degradation of the collagen matrix at the fourth week. By six weeks there was formation of basement membrane with complete re-epithelialization of gingiva. Traces of ADM were also seen after 10 weeks.^27,28^ The present systematic review is consistent with a study by Wei et al.^29^ The study reported that an increase in the width of attached gingiva was observed with SCTG than with ADM graft, which could be attributed to the considerable shrinkage of ADM during the healing phase. SCTG harvested from the palate resulted in more postoperative pain, discomfort and ulceration of the flap because of the more extensive procedure. Hence, ADM can be used as an alternative to avoid multiple tissue harvesting, morbidity, and patient discomfort.^30,31^

 In the present systematic review, both SCTG and ADM were equally effective in reducing the gingival recession depth and accomplishing root coverage. Moslemi et al^14^ evaluated root coverage using SCTG and ADM with a follow-up of 5 years. The study reported that mean root coverage was improved using both grafts at 6 months but decreased significantly over 5 years. The relapse of SCTG and ADM was attributed to the improper toothbrushing technique of the participants.^14^

###  Quality of the evidence

 Randomized control trial (RCT) was assessed as low risk of bias. A more patient-centered outcome can be evaluated using a visual analog scale (VAS), a tool to assess the levels of discomfort and pain after different periodontal treatment modalities. This resource can help evaluate various esthetic and functional outcomes of an individual. A recent study using a VAS showed that pain and discomfort were more significant in the control group than in the test group. Postoperative bleeding, as well as soft tissue necrosis, was observed on the donor and recipient sites of the control group.^11,14^ Some studies demonstrated no signs of allergy, infection, or other complications were seen, indicating that ADM was well tolerated and accepted by the participants.^15,16^

 According to Muthuraj et al,^13^ ADM showed better color matching as it provides a scaffold for the ingrowth of native cells from the recipient bed, giving a better esthetic result and inhibits the effect of the underlying connective tissue which influences epithelial cells by secretion of keratinocyte growth factor, reducing melanin pigmentation.

 Some studies applied root modification agents (e.g., tetracycline solution, EDTA, and citric acid) in recession defects. Nevertheless, these RCTs suggested no significant clinical benefit of root conditioning in conjunction with root-coverage procedures.^8,9,19^

 Overall, the results of this systematic review demonstrated a statistically significant reduction in the extent of GR and an associated gain in the clinical attachment level with or without improvements in the width of keratinized tissue in both control (SCTG) and test (ADM) groups. However, ADM has successfully emerged as an alternative for correcting multiple Class I and Class II gingival recession defects for functional and esthetic purposes.

 Further RCTs are required to evaluate primarily in terms of esthetics, relapse, and secondary outcomes between different procedures. The inclusion of the recession defect type should have been balanced, and the difference in the treatment response of Class I and Class II recession sites should be considered. Limited data is available on the long-term follow-up of the patients treated for gingival recession. Meta-analyses could have given a definitive conclusion regarding the superiority of one graft over the other.

## Conclusion

1. Both SCTG and ADM for treating Miller’s Class I and Class II gingival recession can achieve better root coverage and gain in the width of keratinized tissue.
2. ADM may be an alternative treatment in cases where multiple areas of recession are seen, where SCTGs harvested from the palate area would be insufficient.
3. Most cases produced significant gains in the clinical attachment level and width of keratinized tissue with differences in the percentages of CRC and mean root coverage.
4. SCTG might be considered a gold standard for treating recession-type defects; however, the incidence of discomfort and pain was directly related to donor sites of SCTG.

Within this study’s limitations, SCTG and ADM are equally effective for root coverage in Class I and Class II gingival recession. However, it is the clinician’s decision depending upon the clinical skills, patients’ willingness to undergo surgical procedures involving a donor site, and the cost required for the surgery.

## Competing Interests

 No competing interests.

## Consent for Publication

 Not applicable.

## Data Availability Statement

 The data are available upon request from the corresponding author.

## Ethical Approval

 Not applicable.

## Supplementary Files


Supplementary file 1 contains Table S1.
