# Supplementary material for: Root coverage in Miller’s Class I and II gingival recession using acellular dermal matrix and subepithelial connective tissue graft: A systematic review
Source: J Adv Periodontol Implant Dent. 2025 Jan 20;17(3):126–31. doi: 10.34172/japid.025.3398 (PMC12501474; doi:10.34172/japid.025.3398)
Supplement: Supplementary file 1 — contains Table S1. [file japid-17-126-s001.pdf]

Khan et al, *J Adv Periodontol Implant Dent.* 2025;17(2):126-S1
doi: 10.34172/japid.025.3398
https://japid.tbzmed.ac.ir

Supplementary file 1

Table S1. Characteristics of studies included in the systematic review

	Study	Study design	Study duration	Participants	Parameters	Surgical method	Postoperative care/instructions	Results	Conclusion
1.	**Gholami GA et al. (2013)[8]**	A split-mouth randomized clinical trial Parallel design clinical trial.	6 months	Sixteen patients, each contributing at least 1 pair of Miller's class I or II /ns	Probing depth; height ; (CAL); KTW using acrylic stent	**Test group:** ADM graft (Alloderm, life cell, Branch, NJ) + double papillary flap **Control group:** received the sub-pedicle CTG+ double papillary flap	Periodontal dressing was given. Acetaminophen tablets 325 mg tid for 7 days Amoxicillin capsules (500 mg tid) for 7 days. Suture removal	Probing depth P=0.860 ; (CAL) P=0.711; KTW P=0.12	There were no statistically significant differences between the test and control groups in terms of recession reduction, clinical attachment gain, and reduction in probing depth.

						vertical releasing incision given;	after 10 days 0.12% chlorhexidine digluconate solution 3 times daily for		The control group had a statistically significant increased area of KT after 6

						Root conditioning with 50 mg/ml tetracycline solution	10 days. mechanical tooth cleaning of the treated areas using a soft toothbrush with a modified Stillman technique.		months compared to the test group. Both ADM and SCTG are effective in root coverage ADM can be used as a substitute for root coverage
2.	**Gürlek et al.(2019)**[9]	A split-mouth randomized clinical trial	18 months	Twelve participants; Age: >18 years old l Miller class I or II	(PD;RD;KTW using a periodontal probe (UNC 15 Probe, Hu-Friedy, Chicago, Illinois) and CAL was calculated as the sum of PD and RD	**Test group:** XADM (Mucoderm, Botiss Gmbh, Berlin, Germany)+ MCAF; **Control group:**	No periodontal dressing used; Nonsteroidal anti-inflammatory medication Twice daily 5 days; Suture removal on day 14	CRC month, the difference between the groups was not statistical	Recession depth; Both ADM and SCTG are effective in reduction of recession depth

						connective tissue graft (CTG)+MC AF; No vertical releasing incision given; Root conditioning with 24% EDTA		ly significant (P>0.05) KTW (P=0.009), PD (P=0.027), RD (P=0.044),	CRC percentage Both ADM and SCTG are effective in root coverage the difference between the groups was statistically non significant.<br><br>KTW Increased in the control group than test group at 18 months<br><br>PD, RD were higher in the

									test group and control group
3.	**Jenabian et al.(2020)[10]**	Randomize d double-blind controlled split-mouth study.	6 months	Nine participants Age:> 18 years	GRD; PPD;CAL;KTW; GT; Using Michigan "O" probe Esthetic index	**Test group**: ADM(Cenod erm,Tissue Regeneration Corporation, Iran) + CAF,<br><br>**Control group:** (SCTG + CAF) Vertical releasing incisions given; No root conditioning	Periodontal dressing given Non-steroidal anti-inflammatory ory (Ibuprofen 400mg, Q.I.D for seven days) Systemic antibiotics (penicillin VK 500 mg Q.I.D for seven days) 0.12% chlorhexidine gluconate Suture removal after 14 days	GRD, P=0.40 the thickness of attached gingiva P=0.17, (ppd), P=0.86 CAL P=0.19; KTW P=0.06 GT P=0.42 percentage average root coverage was	percentage average root coverage was less in the test group than the control group significantly, improved PD, RD, and KTW CAL were in the test group than the control group

								p=0.009	
4.	**Kleber Suzuki et al.(2020)[11]**	split-mouth, double-masked, randomized, controlled clinical trial.		Eighteen adult patients (9 males and 9 females, Age: 24 to 50 years; mean age, 34.5 ± 7.5) type 1	(PD);CAL); (GR);(KTH); (KTT)	**Test group:** XADM (Mucoderm Botiss Dental Berlin, Berlin, Germany) + eCAF<br><br>**Control group:** SCTG+ eCAF (partial-thickness flap); vertical releasing incision given;	Non-steroidal anti-inflammatory ibuprofen (600 mg) three times daily for 5 days, and dipyrone sodium (500 mg) four times daily for 3 days Amoxicillin (500 mg) three times daily for 7 days—0.12% chlorhexidine gluconate solution mouthwash	RH P=0.428: RW P=0.141; KTT P=0.1934	Thepresent clinical results showed no significant differencesin the efficacy of ADM and sCTG in the treatment of GR defects. There was no significant difference in the clinical parameters measured at the 3- and 6- 6-month intervals.

						Root conditioning with 24% EDTA	twice a day for the first 15 days; suture removal after 7 (palate) and 15 (recipient area) days and patients were instructed to clean the surgical area with cotton swabs soaked in chlorhexidine solution twice a day for 15 days .		
5.	**Kroiss et al.(2019)**[12]	Controlled clinical trail	Five years	Thirty-two Age >18 years	(GR); Probing pocket depth (PPD);	ADM (Puros dermis, Zimmer	Non-steroidal anti-inflammat	(GR) P=0.015,	The present clinical results showed no

		Parallel design		Miller Class I or II recession defects	(CAL); Width of keratinized tissue (KTW);	Dental) + CAF;<br><br>SCTG single-incision technique described by Hürzeler and Weng) + CAF;<br><br>No Vertical releasing incisions given; No root conditioning	ory (Ibuprofen 400mg 0.12% chlorhexidine gluconate for 14 days Suture removal after 14 days; mechanical tooth cleaning of the treated areas using a soft toothbrush using the roll technique	Probing pocket depth (PPD) P=0.762, (CAL) P=0.512, width of keratinized tissue (KTW) P=0.678 Gingival thickness (GT) P=0.498	significant differences in the efficacy of ADM and sCTG there was no significant difference in the clinical parameters measured at 6-month interval and five years. GR defects, there was a significant difference in the clinical parameters measured at 6-month intervals

									in CTGS but on long-term follow-up there was no significant difference between the two groups.
6.	**Muthurj et al.(2020)**[13]	randomized split-mouth clinical study, which comprised	6 months	15 systemically healthy patients (8 males and 7 females) with an age range of 23–42 years (mean ± standard deviation: 29.67 ± 3.26 years) who had thirty Miller's Class I recession.	depth (GRD); probing pocket depth (PPD); (CAL); width of keratinized tissue (KTW)	**Test group:** ADM +CAF<br><br>**Control group:** SCTG + CAF vertical releasing incisions given;<br>Root conditioning with 24% ethylenediamine	Periodontal dressing was used. Standard postoperative instructions and medications were given.	depth (GRD) P=0.21 (CAL) P=0.57 apico-coronal width of keratinized tissue (KTW) P=0.002	there were no statistically significant differences in the recorded clinical parameters, such as probing pocket depth, clinical attachment loss, and GR depth. ADM group showed a

						inetetraacetic acid for 3 min			better color match than the SCTG group, while SCTG group achieved more than ADM group.
7.	**Moslemi et al.(2011)**[14]	The present split-mouth randomized controlled clinical trial	5 years	16 Participants Age: 24–45 years (mean age at baseline: 39.4 ± 5.2 years); Miller Class I/II .	Probing depth (PD); Recession depth(RD); Gingival width (GW),	ADM(Alloderm; Life cell, The Woodlands, TX, USA) + pedicle flap; CTG (Langer and Langer technique) + pedicle flap Vertical releasing incision given;	Non-steroidal anti-inflammatory medication was prescribed for pain relief; Chlorhexidine rinse twice daily for 2 weeks; Mechanical tooth cleaning of the treated areas using roll technique with	Probing depth P= 0.08 Recession n depth P=0.153 Gingival width P=0.069	Percentage of root coverage: At 5 years, CRC decreased significantly in both groups: 20% (3 sites) and 13.3% (2 sites) in ADM- and SCTG. Both ADM and SCTG are effective in root coverage

						No root conditioning	a soft toothbrush after 6 weeks. Suture removal not specified	Percentage of root coverage p = 1.000	ADM can be used as a substitute for root coverage<br><br>Recession depth; Both ADM and SCTG are effective in reduction of recession depth. When categorizing relapse as a parameter, 12 sites in the ADM group and 9 sites in the SCTG group showed relapse from 6

									months to 60 months<br><br>Gingival width: Both are effective in increasing gingival width. The mean change of GW from 6 months to 60±2 months was statistically higher in the ADM group than in the SCTG group<br><br>ADM is as effective as SCTG and can be used as a

									substitute in Miller's Class I and Class II
8.	**Fahmy et al. (2018)**[15]		Six months	11 subjects Miller Class I or II recession defects	(GR); (CAL); (KTW.)	**Test group** ADM (Alloderm; Biohorizons, Birmingham, AL) + modified tunnel technique; **Control group** Connective tissue graft using trap door procedure+ modified	Non-steroidal anti-inflammatory drugs: Diclofenac potassium 50 mg tablets (Cataflam 50 mg every 8 h for 5 days, Antibiotics: (Augmentin 1 gm, once every 12 h for 5 days; chlorhexidine HCL (0,12%)	(GR) P=0.511; (CAL) P=0.865; (KTW) P=0.828.	Thepresent clinical results showed no significant differencesin the efficacy of ADM and sCTG in treating GR defects. There was no significant difference in the clinical parameters measured at the

						tunnel technique;<br><br>No Vertical releasing incisions were given;<br>No root conditioning	mouthwash three times daily for 14 days;<br>Sutures were removed after 14 days		baseline and 6-month<br>ADM is as effective as SCTG and can be used as a substitute in Miller's Class I and Class II
9.	**Rakasevi et al. (2020)[16]**	split-mouth, single-center, prospective randomized controlled clinical trial	Twelve months	Twenty participants<br>Age: >18 years old;<br>Type 1 GRs	depth (GRD); width (GRW);KTW;CAL; Probing depth (PD), Mean root coverage	**Test group:**<br><br>XADM (Mucoderm m, Botiss dental GmbH, Berlin, Germany) combined +MCAF	Non-steroidal anti-inflammatory Ibuprofen 600 mg for 7 days T.D.S<br>Systemic antibiotics Amoxicillin, 500 mg T.D.S for 7 Days<br>0.12% chlorhexidine solution twice a	GRD<br>P=0.206<br>GRW<br>P=0.348<br>CAL<br>P=0.884<br>PD<br>P=0.929<br>KTW<br>P=0.922<br>GT<br>P=0.058	No statistically significant differences were observed in all clinical parameters between the treatment groups, 6 and 12 months postoperatively<br>Use of porcine-derived dermal

						**Control group:** connective tissue graft (single incision technique)+MCAF	day, 1 minute, for 3 weeks. Suture removal after 14 days of reinforcement of oral hygiene and mechanical plaque removal	MRC P=0.480 CRC P=0.584	collagen matrix resulted in satisfactory results when compared to SCTG in the treatment of MAGR when used in conjunction with MCAT.
10.	**Renato Maluta, (2021)**[17]	split-mouth design, randomized clinical trial	6 months	Fifteen patients Age: >18 years Miller's class I and II	Probing depth (PD) (GR) (CAL) reduction (GR reduction) Percentage of root coverage (%RC) and frequency of complete root coverage (CRC)	**Test group:** XADM (Mucoderm ®, Botiss Biomaterials, Berlin, Germany) + MCAF	Dexamethasone e 4 mg was prescribed 1 h before the surgical procedure. Amoxicillin 500 mg for 7 days and sodium	Percentage of root coverage (%RC and frequency of complete root	CTG and XDM produced oot coverage, with no statistical difference between groups (paired t-test, P >0.05). higher CRC was described

						**Control Group**: CTG (linear technique) + MCAF; No vertical releasing incision given; No root conditioning	dipyrone 500 mg T.D.S in the first 24 h 0.12% chlorhexidine, twice a day for 14 days; Suture removal after 14 days of surgery.	coverage (CRC) Probing depth (PD) (GR) (CAL) reduction (GR reduction)	for CTG compared to XDM (93.33% and 33.33%, respectively) when just considering the greater recession (McNemar test, p = 0.045).
11.	**Shori et al. (2016)**[18]	Parallel design clinical trial.	6 months	Twenty participants; Age:18 to 50 years (mean 29.7±4.35 years) Millers' Class I or II,	recession depth; (CAL); Mean root coverage	**Test group:** ADM (Alloderm: Life cell, The Woodlands, TX, USA) +CAF; **Control group:**	Periodontal dressing Non-steroidal anti-inflammatory (Ibuprofen + P aracetamol, T.D.S for five days) Systemic	Mean root coverage (REC):(p =0.409) Probing pocket depth	1. Both treatments resulted in a significant reduction in and probing pocket depth and a significant gain in and

						connective tissue graft (CTG)(Trap door approach)+ CAF; vertical incision given; No root conditioning.	antibiotics (Amoxicillin 500 mg T.D.S for seven days) 0.2% chlorhexidine gluconate twice daily, for 4-6 weeks Suture removal not specified	(PPD):(p =0.448) (CAL):(p =0.533) Width of keratinized gingiva (WKG): (p=4.02)	width of keratinized gingiva 2. Mean CAL gains and mean root coverage obtained in the ADM group were greater than the SCTG group, but the difference was not statistically significant. The Mean increase in width of keratinized gingiva was significantly greater in SCTG group

									compared to ADM
12.	**Sumana et al.(2017)[19]**	controlled split-mouth clinical study, single-centered	52 months (median recall period: 22 months)	Ten systemically healthy patients<br>Age: not mentioned<br>Miller's Class I and II GR	GR levels;<br>CAL;<br>width of attached gingiva (AG),	**Test group:** ADM<br><br>**Control group:** SCTG	**Not mentioned**	GR levels P=0.56;<br>CAL P=0.36;<br>width of attached gingiva (AG) P=0.17	Thepresent clinical results showed no significant differences in the efficacy of ADM and sCTGin the treatment of GR defects. There was no significant difference in the clinical parameters measured at the 3- and 6- 6-month intervals

13.	**Taiyeb Ali et al.(2015)[20]**	Parallel design clinical trial.	6 Months	Six patients (three males and three females) Age:: 23–58 years (mean age of the 37.8 years); with Miller class I and II GR	Probing pocket depths (PPD); Clinical attachment loss; keratinized gingiva	**Test group:** ADM+CAF<br><br>**Control group:** Connective tissue graft a (modification of the method described by Langer and Langer)+CA F vertical releasing incision given; Root surface conditioning with tetracycline	**Not mentioned**	Recession n height P=0.097 Recession n width P=0.67 Thicknes s of keratinize d ed gingiva P=0.331 Clinical attachment loss P=0.097	The present clinical results showed no significant differences in the efficacy of ADM and sCTG in the treatment of GR defects; there was no significant difference in the clinical parameters measured at the 3- and 6- month intervals

						solution (125 mg/mL saline			
14.	**Vincent-Bugnas et al. (2021)[21]**	prospective single-center split-mouth randomized study	12 months	12 patients (8 women and 4 men) aged 23 to 55 years (mean, 41.2 ± 10.9 years)	probing depth (PD); KT width; and gingival thickness (GT), using a silicone marker measuring. (CAL) Mean root coverage (MRC);	**Test group:** XADM (Mucoderm ®, Botiss Dental, Straumann Group, Basel, Switzerland) + modified coronally advanced tunnel (MCAT) **Control group:** CTG (single-incision technique described by	Non-steroidal anti-inflammatory (paracetamol, 3 g/day) for 7 days; Antibiotics (Amoxicillin 1,000 mg twice for 7 days); 0.2% chlorhexidine twice daily for 14 days; sutures were removed after 14 days gentle brushing resumed.	probing depth (PD); P=0.875 KTT width; P=0.190 gingival thickness (GT), P=<0.001 (CAL) P=0.007 (MRC); *p*=0.005	(CAL) Mean root coverage (MRC) was significantly improved in the control group when compared with the test group. All the other clinical parameter were improved but not statically significant when compared

						Hürzeler and Weng) + modified coronally advanced tunnel			in both the groups.

Subepithlial connective tissue (SCTG); Alloderm (ADM); Mucoderm (MD); Puros dermis (PDAM); Complete root coverage (CRC); Mean root coverage (MRC); depth (GRD); Keratnized tissue width (KTW); Keratinized tissue thickness (KTT); Clinical attachment loss (CAL); Probing depth (PD)

SUPPLEMENTARY FILE 1
